# A novel diagnostic method based on filter bank theory for fast and accurate detection of thermoacoustic instability

**DOI:** 10.1038/s41598-020-80427-6

**Published:** 2021-02-04

**Authors:** Seongpil Joo, Jongwun Choi, Namkeun Kim, Min Chul Lee

**Affiliations:** 1 Gas Turbine Laboratory, National Research Council of Canada, Ottawa, ON Canada; 2 Department of Mechanical Engineering, Incheon National University, Incheon, 22012 Republic of Korea; 3 Department of Safety Engineering, Incheon National University, Incheon, 22012 Republic of Korea; 4 Research Institute of Engineering and Technology, Incheon National University, Incheon, 22012 Republic of Korea

**Keywords:** Mechanical engineering, Scientific data, Aerospace engineering

## Abstract

This study proposes and analyzes a novel methodology that can effectively detect multi-mode combustion instability (CI) in a gas turbine combustor. The experiment is conducted in a model gas turbine combustor, and dynamic pressure (DP) and flame images are examined during the transition from stable to unstable flame, which is driven by changing fuel compositions. As a powerful technique for early detection of CI in multi-mode as well as in single mode, a new filter bank (FB) method based on spectral analysis of DP is proposed. Sequential processing using a triangular filter with Mel-scaling and a Hamming window is applied to increase the accuracy of the FB method, and the instability criterion is determined by calculating the magnitude of FB components. The performance of the FB method is compared with that of two conventional methods that are based on the root-mean-squared DP and temporal kurtosis. From the results, the FB method shows comparable performance in detection speed, sensitivity, and accuracy with other parameters. In addition, the FB components enable the analysis of various frequencies and multi-mode frequencies. Therefore, the FB method can be considered as an additional prognosis tool to determine the multi-mode CI in a monitoring system for gas turbine combustors.

## Introduction

Recently, most gas turbines have adapted a premixed combustion that can reduce hazardous emissions, such as NO_x_ and unburned hydrocarbons, by decreasing the flame temperature and enhancing the mixing of air and fuel^1–3^. However, this has a high risk of combustion instability (CI) caused by: (1) the lower sustainability of flame anchoring; and (2) the higher probability of coupling between pressure fluctuation and heat release fluctuation in both time and position^4–6^. CIs are a physical phenomenon that occur in reacting flows (e.g., a flame) due to constructive coupling processes between acoustic pressure fluctuations and heat release oscillations in the combustion zone. The CI generally accompanies strong pressure vibration to cause damage to the components of the combustor. Furthermore, the debris detached from the combustor can lead to fatal failures of the downstream parts, such as turbine blades and vanes. Thus, CI has been investigated for more than three decades, both theoretically^7–9^ and experimentally^10–15^, to understand its mechanism and devise methods of diagnosis and control.

In view of practical applications of the combustion monitoring of real engines, several diagnostic tools have been introduced to rapidly and accurately detect the onset of CI before it evolves to a significant level^16–23^. Table 1 summarizes representative parameters or analysis methods utilized for CI diagnosis.

**Table 1:** Various parameters or analysis methods for evaluating combustion status.

Parameter or method	Description	References
Growth or damping rate	Growth/damping rate ($\upnu$) is a parameter which quantifies the acoustic-driving/damping capacity in the system of an injector and a chamber so it expresses the tendency toward to amplification/dissipation of combustion instability. It similarly works as a damping coefficient of mass-damper-spring system. If $\upnu >0$, meaning that the driving contribution of the acoustic-flame interaction overcomes the dissipation of acoustic energy and the system stay as unstable. However, if $\upnu <0$, system is stable status	^24–27^
Phase portraits and recurrence plot	Phase portraits are tools to analyze the nonlinear dynamics of a given signal by expressing all states of the physical system in phase space and the recurrence plot is a parameter that visualizes the recurrence of phase space trajectories	^28, 29^
Complex network analysis	The complex networks are derived from the time-series dynamic pressure data that represents the system dynamics using the visibility algorithm. The use of complex network helps to formulate the pattern emerging during the transition in combustion dynamics. Especially, a method combining turbulence network that each fluid element is connected via vortical interaction and machine learning^18^. It can detect a small change in the combustion transition process	^18, 30^
Temporal root mean square	Temporal RMS is a representative value for describing a characteristic or trends of a dynamic pressure signal. When a signal changes in magnitude and sign over time, RMS can indicate the only average magnitude of squared value of the dynamic pressure signal	^31–34^
Temporal kurtosis	Temporal kurtosis is a time-domain statistical parameter, which contains the statistical distribution-quantity of “peakedness” and “tailedness”	^14, 35, 36^

The growth or damping rate ($\upnu$) is a parameter that quantifies the acoustic driving/damping capacity in a system comprised of an injector and a chamber^24–27^. It similarly works as a damping coefficient of the mass–damper–spring system; thus, if $\upnu >0$, the driving contribution of the acoustic–flame interaction overcomes the dissipation of acoustic energy, and the system remains unstable. On the other hand, if $\upnu <0$, the driving of the acoustic–flame interaction is less than the dissipation of acoustic energy, so that the system remains stable. Thus, this parameter has been used when expressing the trend of development or decay of CI. In particular, Yi et al.^26^ investigated a damping ratio for the pressure spectrum using a weighted least mean squares (LMS) algorithm, and they successfully showed that the damping ratio drops rapidly when a nonlinear limit cycle oscillation appears. However, the growth or damping rate has limitations in accuracy because considerable assumptions are required for the combustion process and mechanical system to derive a mathematical model. Another limitation is that real-time monitoring is impossible due to the large amount of computational time for fitting a dynamic pressure (DP) signal to a specific function.

Phase portraits and recurrence plots^28, 29^ are potential analysis tools in nonlinear dynamics, which are applicable to the phenomenon of CI. By expressing all the states of the measured DP data on the phase space, the behavior of the nonlinear combustion phenomenon can be visualized, and the states (e.g., quasi-periodic, chaos, and Hopf bifurcation) can be analyzed. Gopalakrishnan et al.^37^ found a robust indicator for the onset of the subcritical transition of a signal using the Hopf bifurcation method. They successfully validated the critical threshold, called the tipping point, which deceases in recovery rate to equilibrium as a thermoacoustic system approaches the tipping point. In addition, these methods provide significant insight into the nonlinear combustion phenomenon.

Another analysis method, which uses a complex network and represents the system dynamics using a visibility algorithm, has also been proposed. This method uses a turbulence network and machine learning so that it can effectively detect subtle changes in the early period of the transition status. However, there are some drawbacks, in that it cannot suggest the physical meaning of the CI criteria; thus, it is difficult to compare its performance with that of other methods.

Therefore, we propose an analysis method using a filter bank (FB) that can overcome the limitations mentioned above. An FB is an array of bandpass filters that separates the input signal into multiple components, and each component can quantify the average power of a DP signal in the single-frequency subband of the original signal. The concept of an FB is quite similar to that of a bandpass filter designed to separate or remove a specific voice signal from various voices or sounds^38–46^. Although the FB method is advantageous for rapidly detecting not only single-mode CI, but also multi-mode CI, it has not yet been applied to the research field of gas turbine combustion. In the case of single-mode CI, the dynamic response of CI and its frequencies can be categorized as following Table 2.

**Table 2:** Frequency bands of combustion instability and its dynamic response in gas turbine combustor^47^.

Frequency [Hz]	Expected level [psi, pk-pk]	Maximum acceptable level [psi, pk-pk]	Impact
100–135	< 0.5	1	Hardware damage
140–180	< 2	2.5	Initial damage to cross fire tubes a precursor to further hardware damage
2500+	< 0.3	0.3	Levels of 1 psi peak to peak will cause high cycle fatigue, which can quickly crack welds and lead to component hardware failure
10–20	< 2	2	An indication that unit is running too lean and about to flame out

When single-mode CI occurs, the magnitude of CI can be reduced by tuning the combustion or by adjusting operating conditions. On the contrary, when multi-mode CI occurs, frequencies of multiple bands are simultaneously revealed. For example, if low-frequency instability and high-frequency instability occur at the same time with the same magnitude, it can cause failure of the combustor liner while extinguishing the flame, and it is much more severe because the frequencies can synergistically amplify the magnitude of CI. In addition, it is difficult and complicated to control several CI frequencies at once. Therefore, the multi-mode CI can be more severe than the single-mode CI in practical gas turbine combustors.

To verify the feature of multi-mode detection, the FB method is applied to hydrogen-containing syngas, which is prone to generate high multi-mode CI^48, 49^. Hydrogen-containing fuels have recently gained attention, with significant interest in renewable fuels, such as synthetic gas, synthetic natural gas, and blast furnace gas. These gases are known to exhibit the special phenomenon of high harmonic multi-mode in some compositions of H_2_/CO/CH_4_ synthetic gases^50^, which is definitely differentiated from the single-mode phenomenon occurring during combustion of natural gas or methane.

The performance of the FB method is validated by comparing the accuracy and speed of detection with those of three conventional methods: temporal root mean square (RMS), and temporal kurtosis (TK), for which descriptions are listed in Table 1. Since the RMS is the most standard method of CI diagnostics and is widely applied to commercial engines, RMS is firstly selected as a comparison tool^31–34^. TK is chosen secondly, because it is one of the recent methods that can judge the onset of CI, based not on the magnitude of DP but on the periodicity^14, 35, 36^. That is, since the TK contains information on the statistical quantity of “peakedness” and “tailedness,” the distribution and shape of DP can be used when determining the CI driving or decaying. There are other approaches similar to TK. For example, entropy of energy^36^ and permutation entropy^51, 52^ are recent approaches for CI diagnostics based on the signal distribution. In these methods, TK is widely used in the acoustics and CI detection; thus, it has also been selected as a parameter for comparison. From the comparisons with these methods, the better performance of FBs is verified by providing detailed clues, such as advances in sensitivity of CI detection, shorter calculation time with a simpler algorithm and, consequently, earlier and more precise detection results.

## Experimental and data processing methods

### Model gas turbine combustor

**Figure 1:**
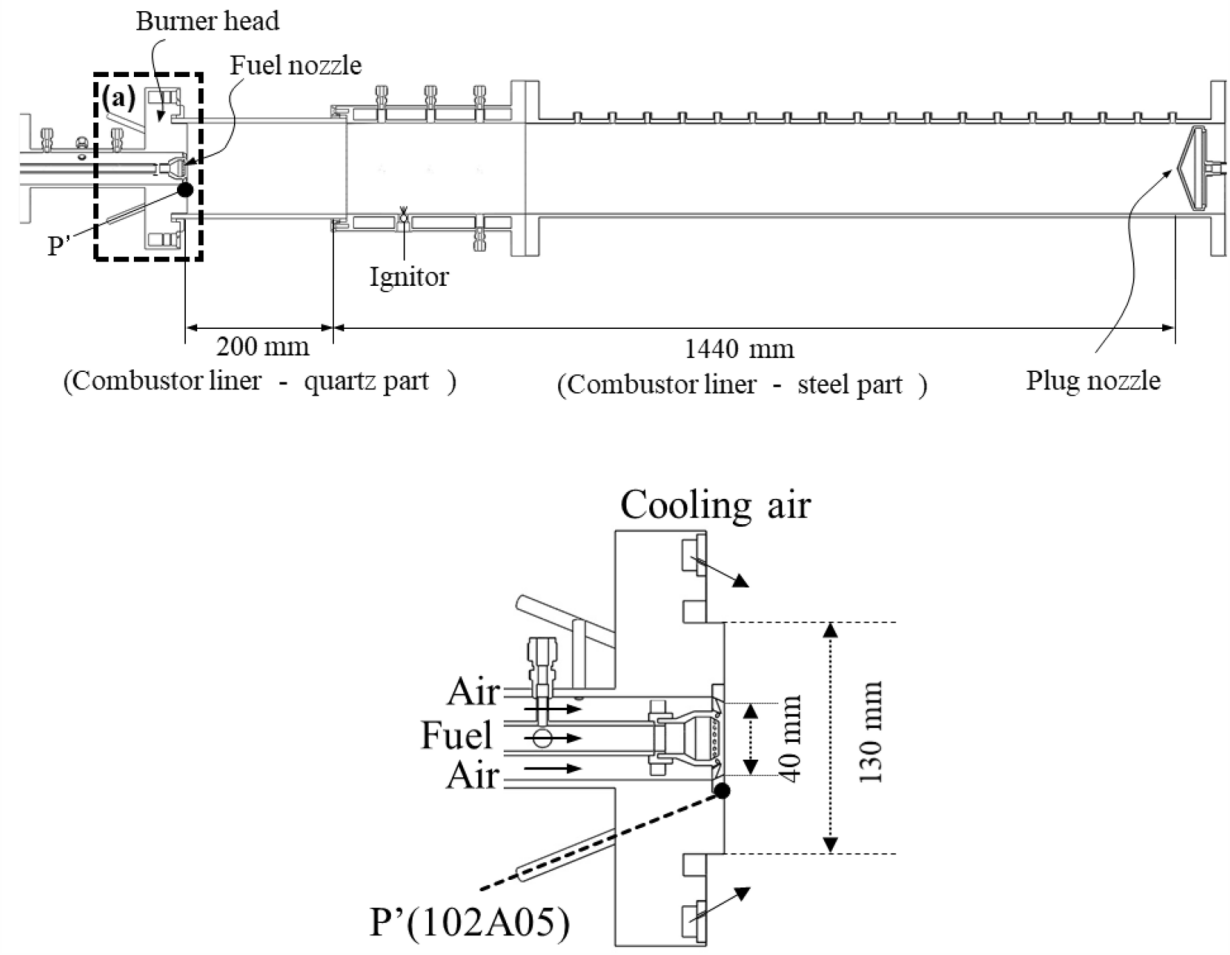
Schematic of the model gas turbine combustor and an enlarged view of the combustor head in Region.

To obtain experimental CI data, a model gas turbine combustor, which generates partially premixed flames, was utilized. As shown in Fig. 1, the fuel mixture and air were supplied in parallel in the coaxial direction through separate injecting holes. At the end of each hole, 14 swirl vanes oriented at 45° with respect to the longitudinal axis of the combustor enabled the two different types of fluids to mix. Despite having a very short mixing length (L_mixing_ = 2.7 mm), it had a strong swirl number; thus, mainly bluish flames appeared similar to a premixed flame.

1 $${S}_{n}=\frac{2}{3}\left[\frac{1-{\left({D}_{swirl\_in}/{D}_{swirl\_out}\right)}^{3}}{1-{\left({D}_{swirl\_in}/{D}_{swirl\_out}\right)}^{2}}\right] \tan \upphi$$

where D_swirl_in_ and D_swirl_out_ are the inner and outer diameters of the swirl vane; and ф is the swirl vane angle. From Eq. (1), the swirl number is calculated to be 0.83^53^.

This combustor mainly generates a longitudinal CI mode rather than a radial or tangential mode because the combustor length from the dump plane to the plug nozzle (L_c_ = 1440 mm) is relatively longer than the radius of the combustor. The dump plane and plug nozzle can be assumed to form acoustically-closed boundary conditions.

2 $$f=\frac{n \cdot c}{2 \cdot {L}_{c}}, \,\,\, n=1, 2, 3, \cdots , c =\sqrt{\gamma \cdot R \cdot {T}_{c}}$$

where $\gamma$ is the adiabatic index; R is the gas constant; L_c_ is the combustor length; T_c_ is the mean temperature in the combustor; and c is the speed of sound in the combustion zone. Using Eq. (2), the frequency of the fundamental longitudinal mode is calculated to be 248 Hz^54^. As *n* changes in Eq. (2), the CI can have other higher modal frequencies. Furthermore, several modes can occur simultaneously in a combustor.

### High-speed measurements for CI observation

To investigate the fast periodic fluctuation of CI, the DP was measured at a rate of 16,000 Hz using a DP sensor (model 102A05, PCB Piezotronics, US) on the dump plane closest to the flame. The DP data were recorded for 3.0 s, which was sufficient to investigate the transition of the DP characteristics from the stable state to the unstable state.

An intensified charge-coupled device camera (model: PI-Max2; 1024 × 1024 pixels; Teledyne Princeton Instruments, US) was also used to observe the flame variation with respect to the fuel composition. An OH bandpass filter (307 ± 10 nm) was installed on the camera to obtain chemiluminescent signals of the OH radical. As the OH chemiluminescent signals represented the heat release characteristics of the combustion zone, this information was used to identify the structural characteristics of the flame.

### Experimental conditions

Table 3 lists the experimental conditions. In this study, highly pure fuels of hydrogen (H_2_) and methane (CH_4_) (purity: H_2_ > 99.5 mol%, CH_4_ > 99.5 mol%) were used to ensure the reliability of the experiment. The fuel and air flow rates were controlled by mass flow controllers (for air: model F-206BI, Bronkhorst High-Tech, Netherlands; for fuel: Porter-200, Parker Hannifin Corp., US). The combustion air was supplied at a flow rate of 1100 SLPM (0.02 kg/s) at a temperature of 200 ± 3 °C. The heat input was varied at 40 kW and 50 kW based on the lower heating value (LHV) of each fuel species. The equivalence ratio was fixed at 0.55. The pressure in the combustor remained almost constant at 1.1–1.2 barg while the fuel compositions of H_2_, CH_4_, and the heat inputs were changed to find the stable and unstable regions. Specifically, the H_2_ ratio was defined as the ratio of the volumetric flow rate of H_2_ to the sum of those of H_2_ and CH_4_. The H_2_ ratio was varied from 0 to 100% with an increment of 12.5%.

**Table 3:** Experimental test conditions.

Parameter	Value
Fuel	H_2_, CH_4_
Air flow rate	1100 SLPM (0.18 kg/s),
Air temperature	200 °C (= 473 K)
Heat input (LHV)	40 kW, 50 kW
Equivalence ratio	0.55
H_2_ ratio [$\frac{{H}_{2}}{{H}_{2}+{CH}_{4}}$ $\frac{{\text{H}}_{2}}{{\text{H}}_{2}+{\text{CH}}_{4}}$]	0–100% (span of 12.5% by volumetric ratio)
Combustor length	1440 mm

### Data processing method

Before introducing the details of the FB method for CI analysis, the TK method, which will be compared with FB, is briefly reviewed.

#### Temporal kurtosis

In order to quantify the instantaneous “peakedness” and “tailedness” of the statistical distribution of a given signal, the TK is calculated as follows:

3 $$TK=\frac{\frac{1}{n}{\sum }_{i=1}^{n}{\left({p}_{i}-\stackrel{-}{p}\right)}^{4}}{{\left[\frac{1}{n}{\sum }_{i=1}^{n}{\left({p}_{i}-\stackrel{-}{p}\right)}^{2}\right]}^{2}}$$

where *n* is the number of DP data values for a duration of given time (T); *p*_*i*_ is the instantaneous DP signal at time *t* = *t*_*i*_ (*i* = 1, 2 …, n); and $\stackrel{-}{p}$ is the mean DP. The TK has the theoretical value according to the given data properties. That is, whereas the TK for a random Gaussian distribution (i.e., a stable status in a combustion) is 3.0, the TK for a sinusoidal signal (i.e., an unstable status in a combustion) is 1.5. In a practical combustor, however, the DP signal of a stable status can be not a perfect Gaussian distribution, which means that the TK cannot be 3.0 in spite of the stable combustion status. To avoid this shortcoming, previous studies used the average value of several TKs through a linear regression rather than the single TK value^35^. On the contrary, it should be noted that the FB method suggested in the current study can assess the combustion status with only the single value at a specific moment.

#### Filter bank

**Figure 2:**
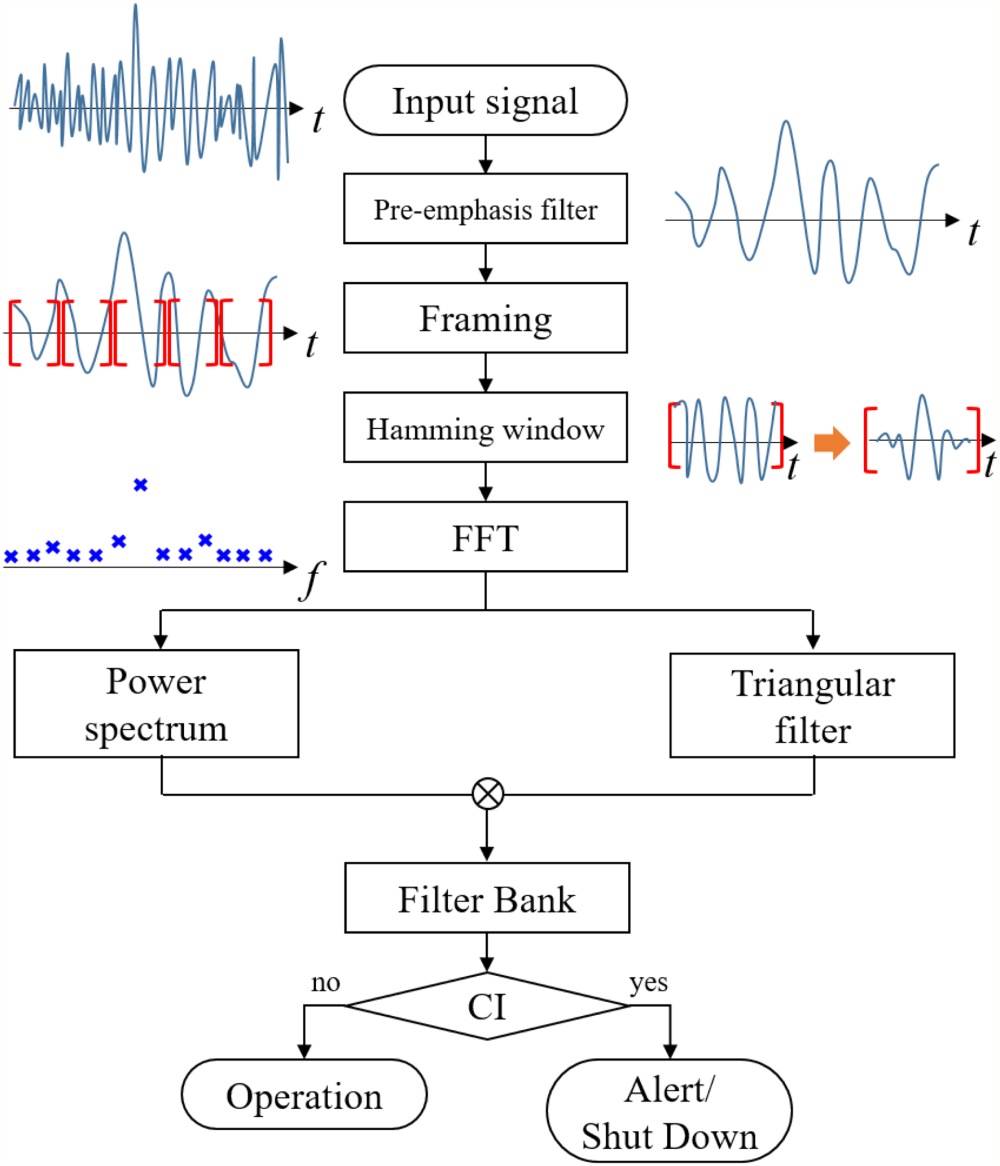
Flowchart of the FB processing method for CI diagnosis.

The FB is a signal processing method based on an array of bandpass filters. It can detect the characteristics of various frequency bands by generating subband filters and multiplying them with the power spectrum of each frequency obtained from a given dataset. In this study, the FB of DP signals was calculated and used as a CI precursor. As the first step, the noise of an input signal, DP data, is removed by a pre-emphasis filter. As the next step, the signal set is divided into several subsets by a time framing process in order to increase the time resolution. Then, a Hamming window is applied to each subset to reduce the leakage error. As the next step, the fast Fourier transform (FFT) is performed for signals filtered by the Hamming window. In this process, the signal domain changes from time to frequency. Next, the FFT signals are used to calculate the power spectrum that represents the energy of the signal at the corresponding frequency. The power spectrum at each frequency is obtained by the squared magnitude of the FFT result. Independently of the power spectrum calculation, the frequency range obtained by the FFT is divided into sub-frequency bands using a triangular filter. As the final step, the results of two independent processes, power spectrum and triangular filtering, are multiplied to obtain the FB component. Figure 2 summarizes the FB calculation process. Among all the steps, details for three significant steps (Hamming window, triangular filter, and FB calculation) are described in the following subsections.

#### Hamming window

**Figure 3:**
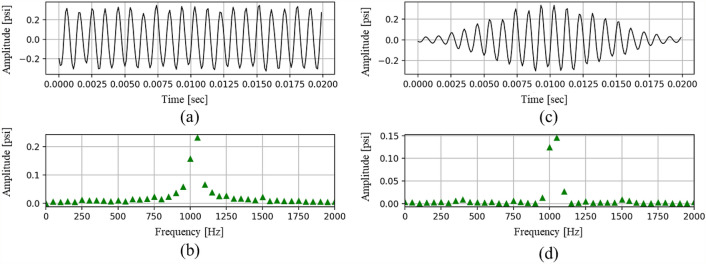
While each row distinguishes the calculation domain as time and frequency, each column represents the DP data before and after a Hamming window. Raw dynamic pressure (**a**) before the Hamming window in the time domain, (**b**) before the Hamming window in the frequency domain (i.e., FFT results), (**c**) after the Hamming window in the time domain, and (**d**) after the Hamming window in the frequency domain (i.e., FFT results).

DP signals without any windowing process induce discontinuous FFT results at the beginning and end of the signal, which is termed as “leakage error.” A Hamming window can prevent this error in the FFT analysis^55^. When a Hamming window is applied on raw DP signals, it can alleviate the discontinuity at both ends of the frame and attenuate the magnitude of the sidelobes. Figure 3 shows the FFT results before and after applying the Hamming window to the raw signal. While the first row (Fig. 3a,c) represents the raw data in the time domain, the second row (Fig. 3b,d) indicates the FFT results in the frequency domain. In addition, whereas the first column (Fig. 3a,b) describe the results before the Hamming window, the second column (Fig. 3c,d) represents the results after the Hamming window. The Hamming window is defined by Eq. (4), where *N* is the number of data values in a frame.

4 $$Hamming\; window, w\left(n\right)=0.54-0.46\,\,\, \text{cos}\left(2\pi \frac{n}{N}\right) , \,\,\, 0\le n\le N$$

#### Triangular filter

The main purpose of the triangular filter is to divide the frequency range into sub-frequency bands, which can efficiently reflect spectral characteristics in a certain band rather than a specific frequency. In this study, the frequency domain is divided by the Mel-scales in which the frequency resolution is higher at low frequencies than at high frequencies. As the Mel-scaling converts the linear scale into a logarithmic scale in frequency, it enables more detailed examination at the low-frequency bands by making the low-frequency band more sensitive than the high-frequency band. The Mel-scale is defined as

5 $${Mel \text{-}scale{:}\;\; m(n)=2595\times \log}_{10}\left(1+ \frac{f{^{\prime}}\left(n\right)}{700}\right)$$

where *n* is the number of sub-frequency bands, and *f ′* is the modified sub-frequency by the Mel-scaling. The detailed process for the Mel-scaling is as follows:

First, as the DP data are acquired at 16,000 samples per second in the current study, the frequency range of the FFT is from 0 to 8000 Hz. We can obtain two Mel-scaled values by inserting the minimum and maximum frequencies, 0 Hz and 8000 Hz, into Eq. (5), yielding 0 and 2840.

Second, the obtained Mel-scaled range, 0–2840, should be linearly divided into 40 subbands. Here, although the number of subbands can be varied, we select the number as a commonly used one, 40^56^. The linearly divided values are 0, 69.3, 138.5, etc. These calculated values are summarized in Table 4.

**Table 4:** Mel-scale conversion chart.

m(n) in Eq. (5)		*f'(n)* in Eq. (5)		Triangular Filter’s frequency bandwidth
m(n)	Frequency [mels]	*f'(n)*	Frequency [Hz]	*f'(n − 1)* – *f'(n* + *1)* [Hz]
m(0)	0.0	*f″0)*	0.0	–
m(1)	69.3	*f″(1)*	44.4	0–91.6
m(2)	138.5	*f″(2)*	91.6	44.4–141.7
$\vdots$		$\vdots$		$\vdots$
m(39)	2701.5	*f′(39)*	6993.7	6535–7481.4
m(40)	2770.8	*f'(40)*	7481.4	6993.6–8000
m(41)	2840.0	*f'(41)*	8000.0	–

Third, the Mel-scaled values are inserted again into the Eq. (5) to determine the corresponding frequency, *f ′*. Obviously, the frequency values at the boundaries are 0 Hz and 8000 Hz. The other frequency values are summarized in Table 4.

**Figure 4:**
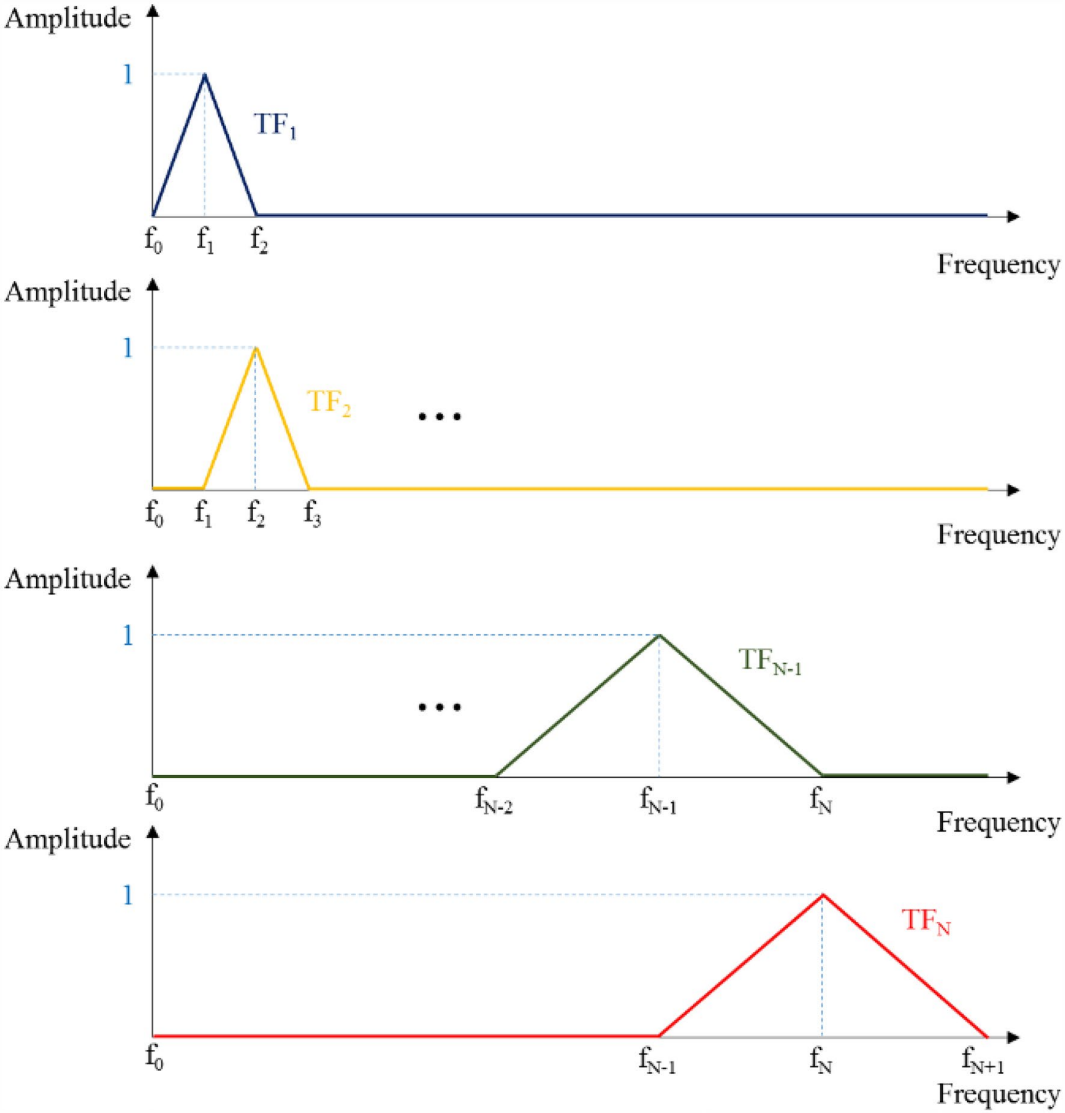
Mechanism of the triangular filter.

Lastly, to construct the triangular filter, three frequencies are used from *f ′*. If the *n*th frequency, *f ′(n)*, is of interest, its filter amplitude is set up to be 1, while the amplitude of *f ′* (*n − *1*)* and *f ′* (*n* + 1) are 0. This process is described in Fig. 4. It should be noted that *f ′* is different than *f* obtained from the FFT. Therefore, there can be several frequencies, *f*, between *f ′* (*n − *1) and *f ′* (*n*), or *f ′* (*n*) and *f ′* (*n* + 1). In this case, the filter amplitude for *f* can be easily calculated by interpolation between 0 and 1, which is expressed as follows:

6 $$TF_{n} \left( f \right) = \left\{ {\begin{array}{ll} 0 \hfill & \quad {f < f'\left( {n - 1} \right)} \hfill \\ {\frac{{f - f^{{'\left( {n - 1} \right)}} }}{{f^{{'\left( n \right)}} - f^{{'\left( {n - 1} \right)}} }}} \hfill & \quad {f'\left( {n - 1} \right) \le f < f^{\prime}\left( n \right)} \hfill \\ 1 \hfill & \quad {~f = f'\left( n \right)} \hfill \\ {\frac{{f'\left( {n + 1} \right) - f}}{{f'\left( {n + 1} \right) - f'\left( n \right)}}} \hfill & \quad {~f'\left( n \right) < f \le f'\left( {n + 1} \right)} \hfill \\ 0 \hfill & \quad {f > f'\left( {n + 1} \right)} \hfill \\ \end{array} } \right.$$

where ${TF}_{n}\left(f\right)$ is the magnitude of the *n*th triangular filter according to frequency *f*, *n* is the number of triangular filters, and *f ′ *(*n*) is the calculated frequency with Mel-scaling using Eq. (6).

#### FB components

**Figure 5:**
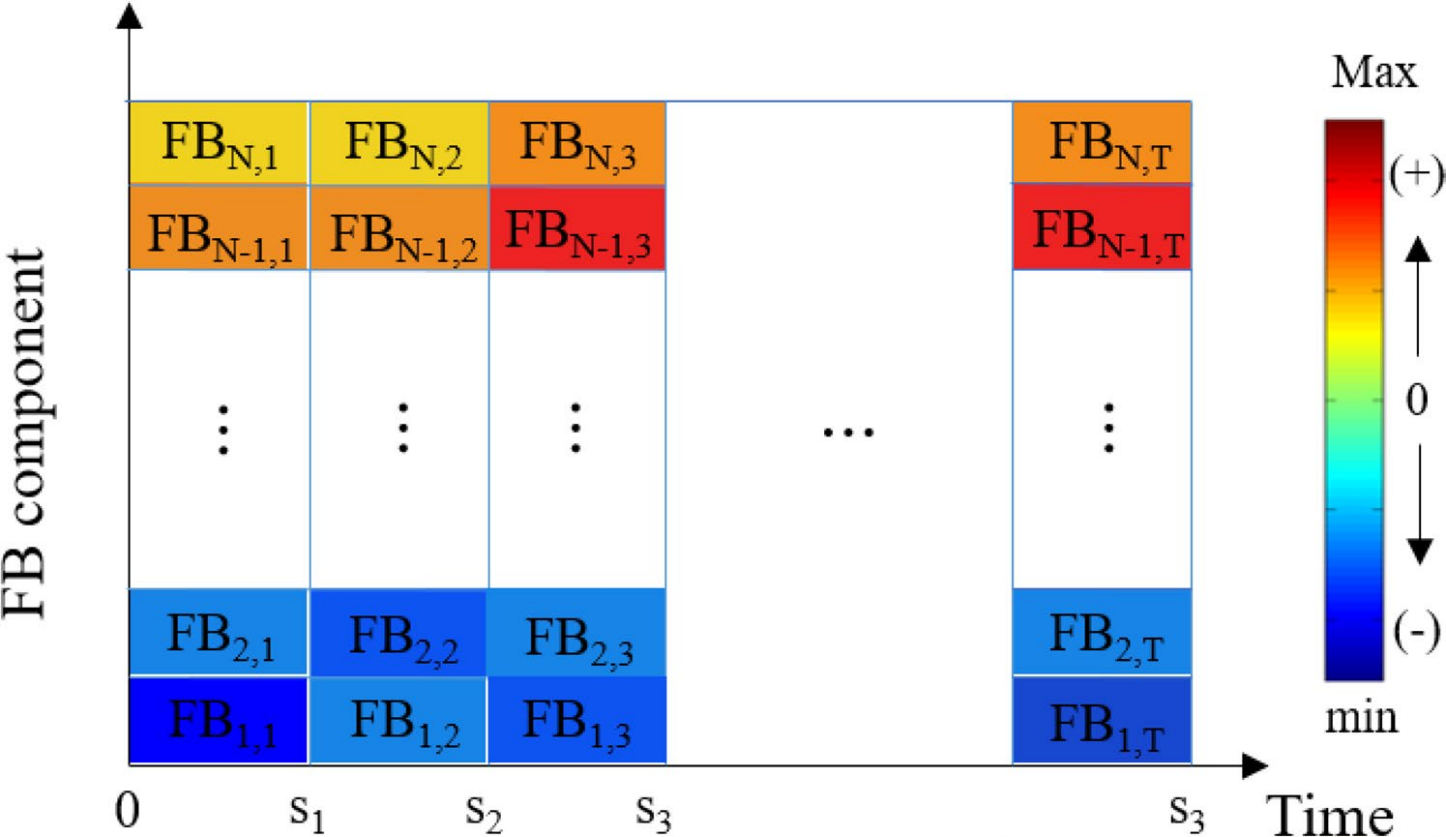
Matrix of the magnitude of the FB components.

The FB component at a specific time interval, *t*, in the *n*th bandwidth is obtained by multiplying the result of the power spectrum and the *n*th triangular filter, as shown in Eq. (7). The calculated FB components are represented by the decibel scale to reflect changes in DP in the state of CI. It should be noted that the FB components are described in the time–frequency domain. In other words, whereas the time interval of interest is represented on the *x-*axis, the sub-frequencies are indicated on the *y-*axis. Therefore, another independent axis is needed to describe the magnitude of the FB components. In this study, the magnitude is represented by color bars rather than values on an additional axis, i.e., the *z-*axis. Figure 5 shows the FB results described above.

7 $$F{B}_{n,t}=\sum (P{S}_{n,t}\times T{F}_{n,t})$$

where *PS*_*n,t*_ and *TF*_*n,t*_ are the power spectrum and triangular filter components for the *n*th subband of the FB at time *t*.

As the FB method can display the intensity of CI for every individual subband with respect to time, it is possible to analyze various frequencies simultaneously (i.e., multi-mode CI). In this study, these particular characteristics of FB methods have been validated by applying the transition combustion process from a stable state to an unstable state.

## Results and discussion

### Combustion instability characteristics of H_2_/CH_4_ flames

To grasp the onset of CI from DP signals during the CI transition process, it is necessary to understand the combustion characteristics of the gas turbine combustor for various compositions. In this study, the criterion of the onset of CI is set to 0.3 psi as the RMS value of the DP signal after the FFT, which is equivalent to 2% of the static pressure of the combustion chamber. This criterion is the most rigorous in comparison to industrial gas turbines, for which the criterion is generally set to 3–5% of static pressure (SP) of the combustor^32^ (Dawson et al.: 2%^31^; Lieuwen et al.: 3%^32^; Rodriguez-Martinez et al.: 5%^33^; Lee et al.: 10%^34^).

Table 5 lists the frequencies, corresponding harmonic modes, and the amplitudes of DP according to the H_2_/CH_4_ ratio and heat input. The frequency and its amplitude corresponding to CI are highlighted in bold. As the ratio of H_2_ increases, the CI frequency is shifted to a higher mode. Therefore, transitions of the harmonic frequencies from the 3rd mode frequency for 12.5–25% H_2_ via the 4th mode frequency for 37.5–62.5% H_2_ to the 6th mode frequency for 75–100% H_2_ are observed. In addition, as the heat input increases, the amplitude of DP increases due to the rise of heat release in the combustion zone. This consequently increases the probability of coupling between fluctuations of the pressure and the heat release rate in the combustion zone^54^, namely, the Rayleigh index.

**Table 5:** Frequencies and corresponding amplitudes of combustion instability with respect to the H_2_:CH_4_ ratio and heat input.

H_2_:CH_4_	40 kW		50 kW	
	Frequency [Hz]	Amplitude [psi]	Frequency [Hz]	Amplitude [psi]
0:100	62	0.016	594	0.01
12.5:87.5	763	0.017	**788 (3rd mode)**	**0.445**
25:75	763	0.098	804	0.012
37.5:62.5	**1015 (4th mode)**	**0.753**	**1069 (4th mode)**	**0.727**
50:50	**1026 (4th mode)**	**0.586**	**1070 (4th mode)**	**0.912**
62.5:37.5	**1029 (4th mode)**	**0.603**	**1078 (4th mode)**	**0.857**
75:25	**1496 (6th mode)**	**0.319**	**1590 (6th mode)**	**0.802**
87.5:12.5	710	0.006	**1600 (6th mode)**	**0.541**
100:0	702	0.008	1635	0.008

As listed in Table 6 and highlighted in Table 5, two stable cases (Case 1 and Case 3) and two unstable cases (Case 2 and Case 4) are selected as representative cases using predetermined information for stable and unstable conditions to compare the effectiveness of the two different methods for CI assessment: TK and FB. In addition, these cases are used to calculate the detection time in the transition period when changing the fuel composition (e.g., from Case 1 to Case 2 or from Case 3 to Case 4).

**Table 6:** Case study data sets.

#	Status	Fuel composition	Heat input ( kW)
Case 1	Stable	H_2_:CH_4_ = 87.5:12.5	40
Case 2	Unstable	H_2_:CH_4_ = 75:25	
Case 3	Stable	H_2_:CH_4_ = 100:0	50
Case 4	Unstable	H_2_:CH_4_ = 87.5:12.5	

### FB analysis of steady-state CI

**Figure 6:**
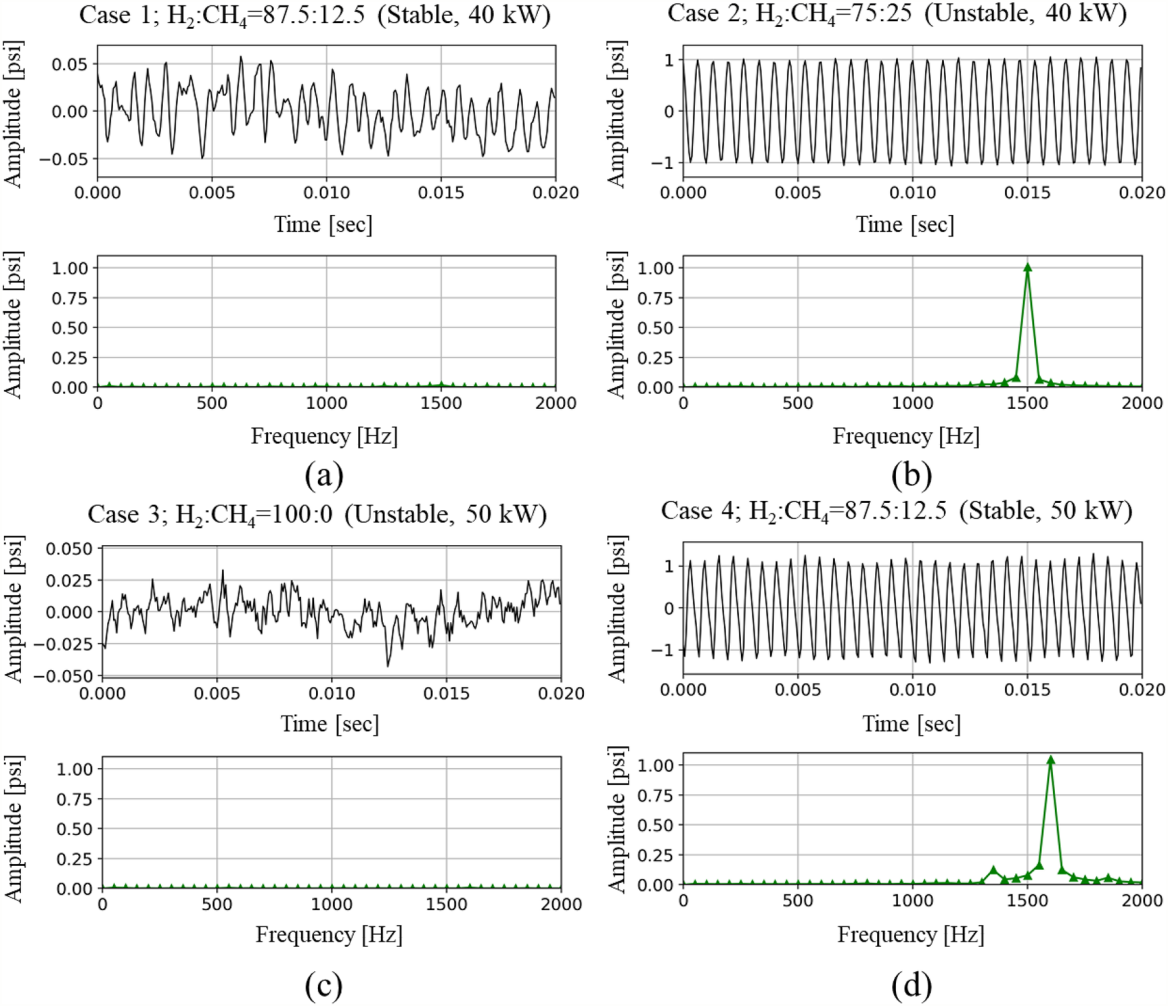
Dynamic pressure signal and FFT results (stable, unstable) for each fuel composition. (**a**) Case 1, (**b**) Case 2, (**c**) Case 3, and (**d**) Case 4.

As shown in Fig. 6, the time series of the DP and its FFT results for Cases 1–4 are examined to determine the optimal size of the subband. In Cases 1 and 3 (i.e., stable condition), the peak-to-peak amplitude of the time series DP is under 0.3 psi and the amplitudes in the frequency domain are also low and show no peak over the entire frequency range. However, in Cases 2 and 4 (i.e., unstable condition), the peak-to-peak amplitude in the time domain is over 0.3 psi and the amplitudes in the frequency domain are also quite high and show only one high peak at a specific frequency. As data acquired during 0.02 s sufficiently represents the main frequencies as well as periodic features of the DP, the size of the subband was set as 0.02 s.

**Figure 7:**
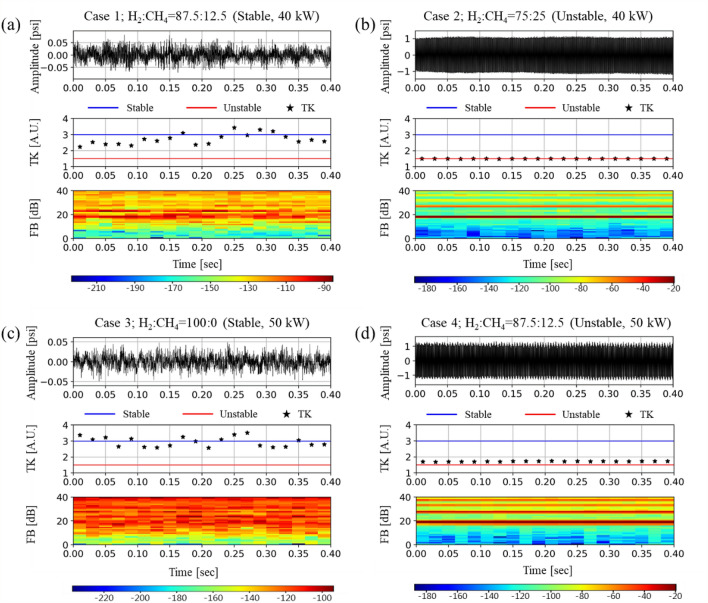
Dynamic pressure signal, temporal kurtosis, and FB components for each fuel composition. (**a**) FB_stable_ = 23rd (− 88 dB), (**b**) FB_unstable_ = 19th (− 20 dB), (**c**) FB_unstable_ = 24th (− 94 dB), and (**d**) FB_stable_ = 19th (− 20 dB).

Figure 7 shows the results of FB analysis together with the time series of the DP and TK analysis results for Cases 1–4. The FB components are calculated from 6400 DP signal samples acquired for 0.4 s after applying the power spectrum of the FFTs and triangular filter. Subsequently, the criteria of FB components for stable and unstable status have been calculated from 16,000 DP signals acquired for 1.0 s in stable and unstable H_2_/CH_4_ fuel compositions, respectively. The maximum and minimum magnitude of the FB components under stable and unstable conditions have been determined as the criterion for CI determination. In this study, “under − 85 dB” and “over − 52 dB” were set as the criterion for stable and an unstable condition, respectively.

The results of FB analysis for Cases 1–4 have been compared with those of the time series of DP and TK analysis, as shown in Fig. 9. In each sub-figure, a – d, the first, second, and third row indicate the time series of the DP, TK analysis, and FB component, respectively. Furthermore, while Fig. 7a,c show the results obtained from the fuel compositions causing steady and stable combustion, Fig. 7b,d represent the results from the fuel compositions causing steady-unstable combustion. In the FB components for Cases 1 and 3 (i.e., the third row in Fig. 7a,c), the maximum values are − 88 dB at the 23rd subband (bandwidth: 2006.13–2360.09 Hz) and − 94 dB at the 24th subband (bandwidth: 2177.67–2554.08 Hz), respectively. As the values satisfy the predetermined stable criterion, i.e., “under − 85 dB,” it can be assessed as a stable condition. However, the maximum FB components for Cases 2 and 4 (i.e*.,* the third row in Fig. 9b,d) are both − 20 dB at the 19th subband (bandwidth: 1450.45–1693.11 Hz). In these cases, as the value satisfies the unstable criterion, i.e., “over − 52 dB,” it can be determined as unstable.

However, if the FB component lies between − 85 and − 52 dB, it is impossible to assess the combustion status. Therefore, a single value criterion for CI assessment must be set up instead of two different values, such as − 85 dB and − 52 dB. To develop the single value criterion, the cut-off time (*t*_*cut-off*_) has been applied to the FB component and used as the new criterion for CI assessment. The *t*_*cut-off*_ value for the FB component has been defined according to the time when the FB component reaches $1/\sqrt{2}$ of the difference between the stable and unstable criteria, which implies that the *t*_*cut-off*_ is − 62 dB (= (− 85 + 52)/$\sqrt{2}$) in this study^57^.

**Figure 8:**
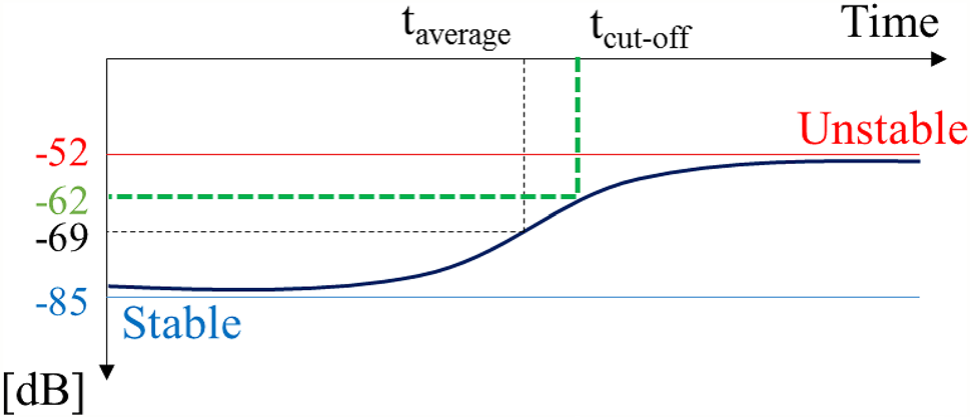
Determination of the cut-off time to obtain early alert for combustion instability.

Figure 8 shows a schematic diagram of *t*_*cut-off*_ when the FB component changes from a stable to an unstable condition. Based on the definition of the cut-off time, − 62 dB (= (− 85 + 52)$/\sqrt{2}$) is considered as the new criterion of CI determination. The cut-off time (*t*_*cut-off*_) detects somewhat later than the average time (*t*_*average*_) of the two stable/unstable reference values. However, the determination of CI occurrence by *t*_*cut-off*_ can be considered as more precise if we consider the possibility of returning to a stable flame during the transition process.

### Performance comparison of TK and FB during transient condition

**Figure 9:**
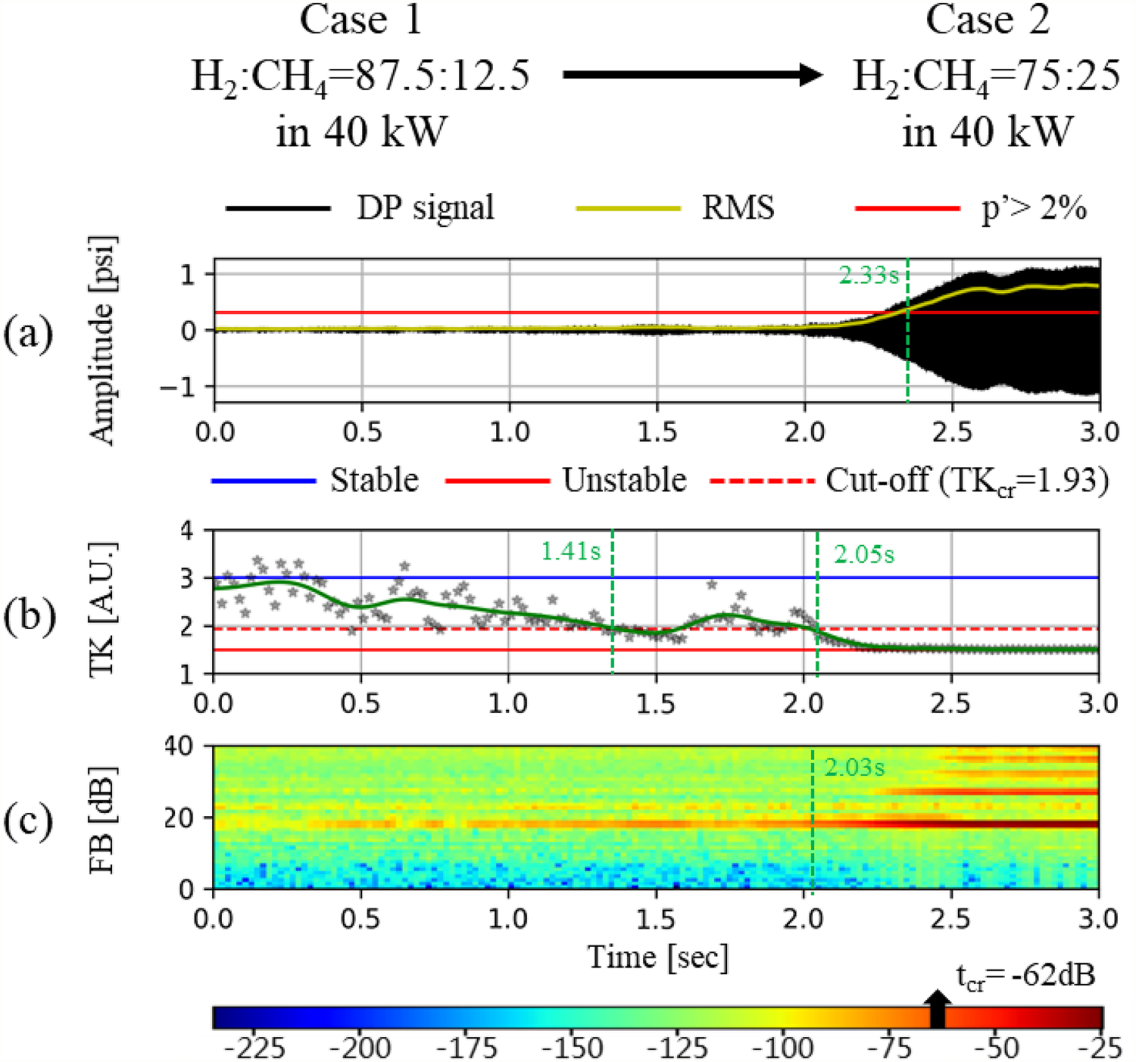
CI analysis results using (**a**) RMS, (**b**) TK, and (**c**) FB for transition from Case 1 to Case 2.

**Figure 10:**
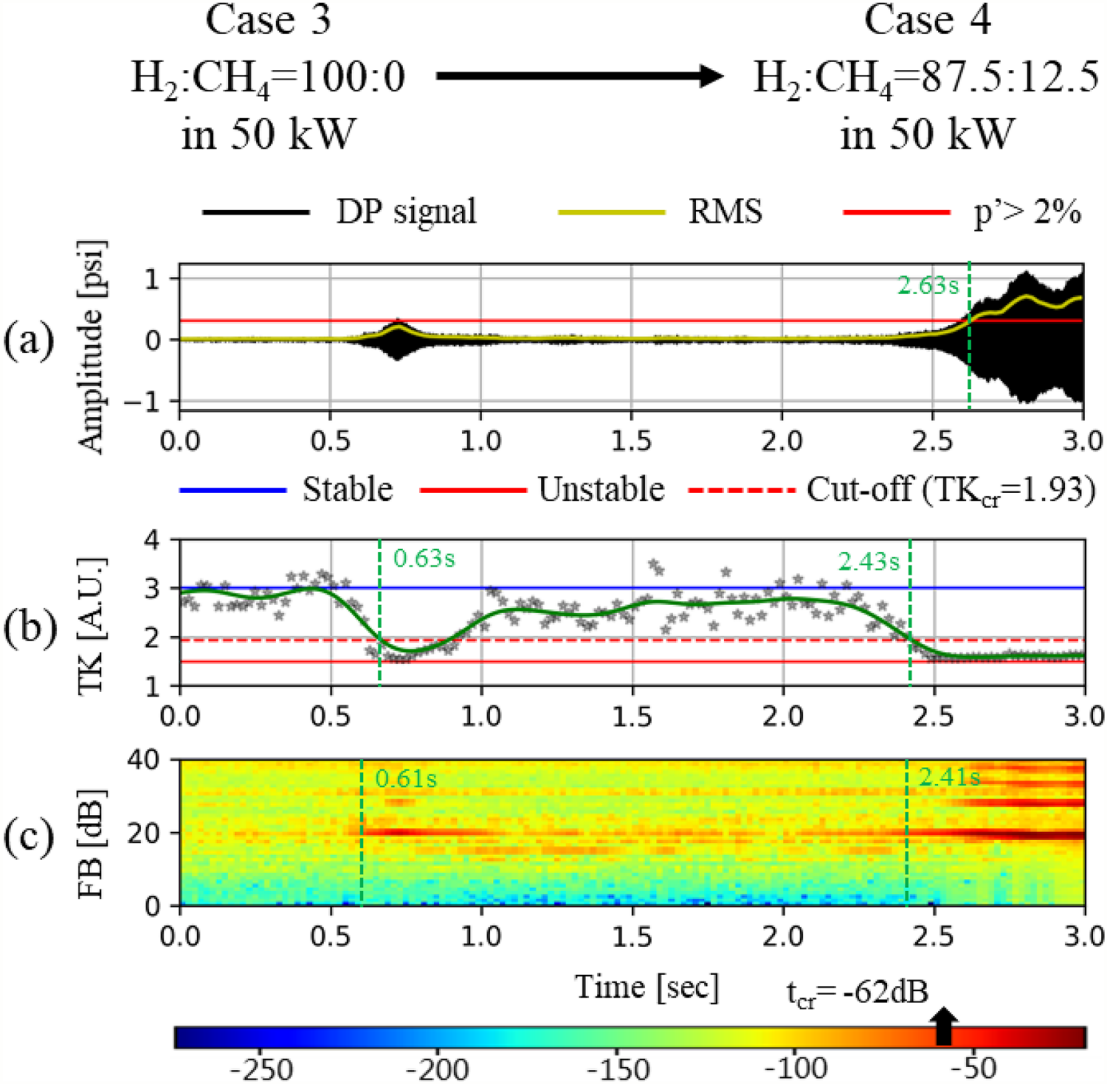
CI analysis results using (**a**) RMS, (**b**) TK, and (**c**) FB for transition from Case 3 to Case 4.

Figures 9a and 10a show the time series DP signals during the CI transition from stable to unstable conditions (Case 1 → Case 2, and Case 3 → Case 4). The signal is acquired for 3.0 s such that 48,000 DP samples are obtained. As previously mentioned, if the RMS of the DP signal exceeds 2% of the static pressure, it can be judged that CI has occurred. Based on the 2% criterion, the CI onset time is determined to be 2.33 s for the transition of Case 1 → Case 2 and 2.63 s for the transition of Case 3 → Case 4.

The CI assessment results using three different methods (i.e., RMS, TK, and FB) in the status transition have been compared in view of the detection time and accuracy, as shown in Figs. 9 and 10. Initially, if we apply the RMS criterion for CI assessment (i.e*.*, exceeding 2% of the static pressure in a combustor), the CI onset time is detected as 2.33 s for the transition of Case 1 → Case 2, and 2.63 s for the transition of Case 3 → Case 4. When the method is changed from RMS to TK, a new criterion for CI assessment has been set as 1.94 (= 3 − 1.5$/\sqrt{2}$) according to the definition of the cut-off time. The detection time from TK is 2.05 s for Case 1 → Case 2, and 2.43 s for Case 3 → Case 4, which is much earlier than for the RMS method. If we use the FB method, the detection time for the two cases is 2.03 and 2.41 s. The FB method shows comparable results for RMS and TK. Although the difference from TK in detection time is as small as 0.4 s, such a small leading time can be very meaningful for the monitoring system operated by automatic electronic control units. Next, in view of the accuracy, the RMS methods detects the CI at 2.33 s and 2.63 s, respectively, for Case 1 → Case 2, and Case 3 → Case 4. However, in Fig. 10, the first peak of pressure is observed at approximately 0.7 s. The RMS method does not detect the first peak, but the TK method does. As shown in Fig. 10b, the TK value is below the criterion of 0.63 s, which implies that TK detects the CI at this time. Such sensitive as well as early detection is the strong aspect of the TK analysis method. However, TK shows disadvantages in that the values at each frequency (i.e., asterisk marks in Figs. 9b and 10b) fluctuate too much. Although linear regression has been applied to TK in order to alleviate the effect of the outlier values, TK shows more fluctuation than RMS or FB, as shown in Figs. 9 and 10.

**Figure 11:**
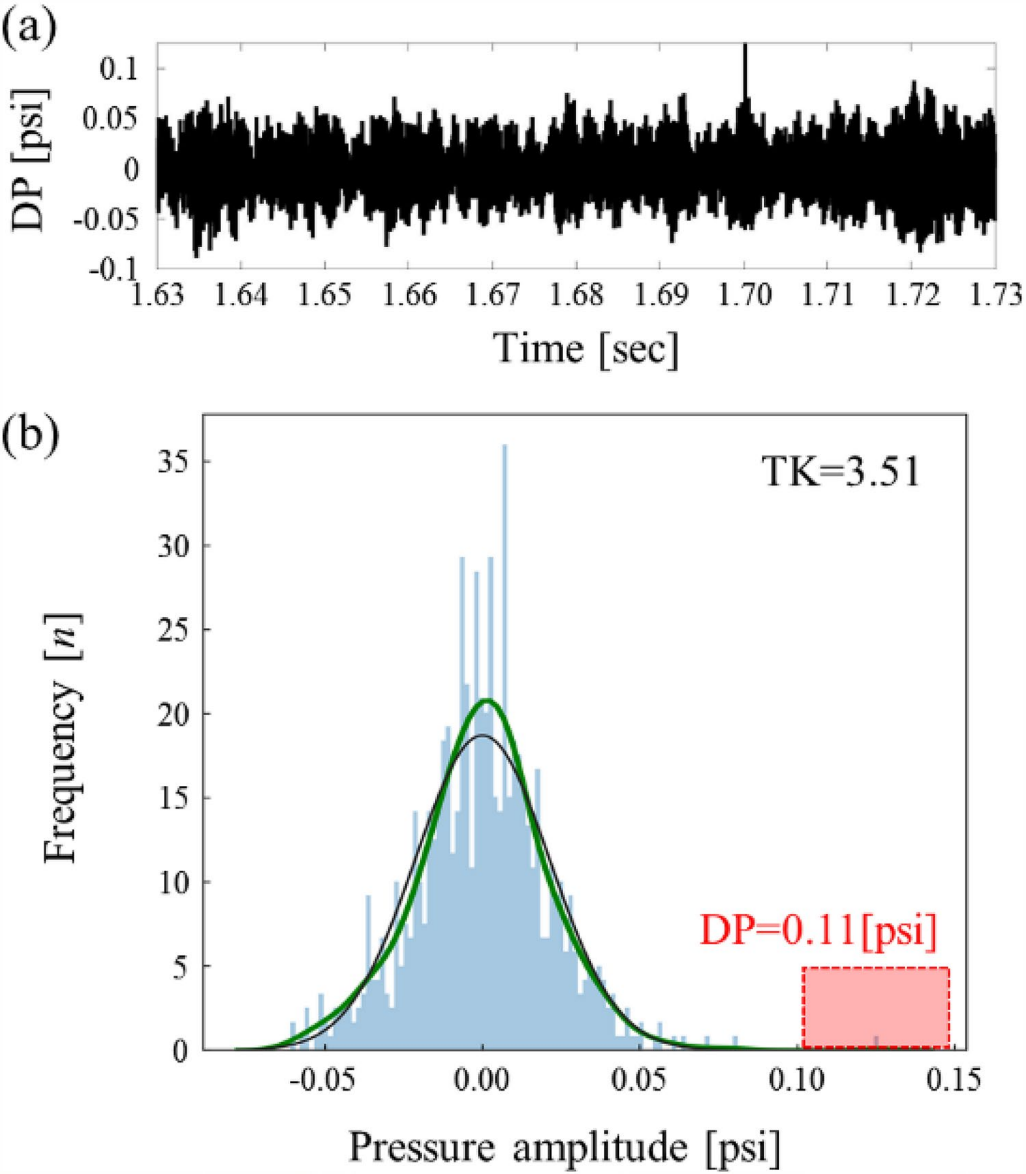
Detailed analysis of the DP signal with an outlier. (**a**) Magnitude (**b**) histogram of DP.

The TK values are 3.37 at 0.22 s in Fig. 9b and 3.51 at 1.68 s in Fig. 10b, respectively, which exceeds the stable reference of 3.0 as the theoretical maximum value of TK in a stable condition. In the frame at 1.68 s in Fig. 10b, the DP signal histogram, as shown in Fig. 11, has a stable normal distribution that is centered at 0.0, but there is an outlier signal that has a magnitude of 0.11 psi. According to the definition of TK, the quadratic term of the numerator with an outlier has a great influence in determining the value of TK. Even though the data points are ordered around the unstable criterion during the CI, several TK values exceed the stable reference of 3.0 during stable conditions. Therefore, the large fluctuation of TK in the stable regime can cause the false-positive detection by approaching the CI criterion of TK.

FB detects not only the 19th subband (bandwidth: 1450.45–1693.11 Hz) but also the other frequencies, such as 27th (bandwidth: 2760.36–3212.98 Hz), as shown in Figs. 9c and  10c. Herein, the second subband frequency is the higher harmonic mode of the 19th subband, which has multi-mode frequencies. However, TK cannot show the various CI frequencies occurring simultaneously because it is performed using only the main lobe of the CI frequency. Therefore, the detection of multi-modes of CI can be ensured only through the FB method.

The drawback of the FB method is that the criterion of FB can differ according to the combustor type and operation conditions because the signal characteristics and frequency bands vary depending on the type of combustor. Furthermore, the criterion also varies according to the sampling rate of the DP signal and frame size. However, if the magnitude of the FB component is initially set up for stable and unstable flames, the FB method can be used as an additional indicator of detection of CI for a monitoring system or combustion tuning procedure.

### Post-processing computation time

Although the detection time of the onset of CI is calculated correctly using each method, it can be an inefficient method if it requires more time for data collection and calculation for post processing. The optimal number of DP signals in the present study is 320, and it takes 20 ms to acquire one frame. The DP data acquired for 3 s is divided into 150 frames to calculate the averaged computation time (t_com_) of post-processing for each frame because the DP data for 1 s consists of the sum of 50 frames.

**Figure 12:**
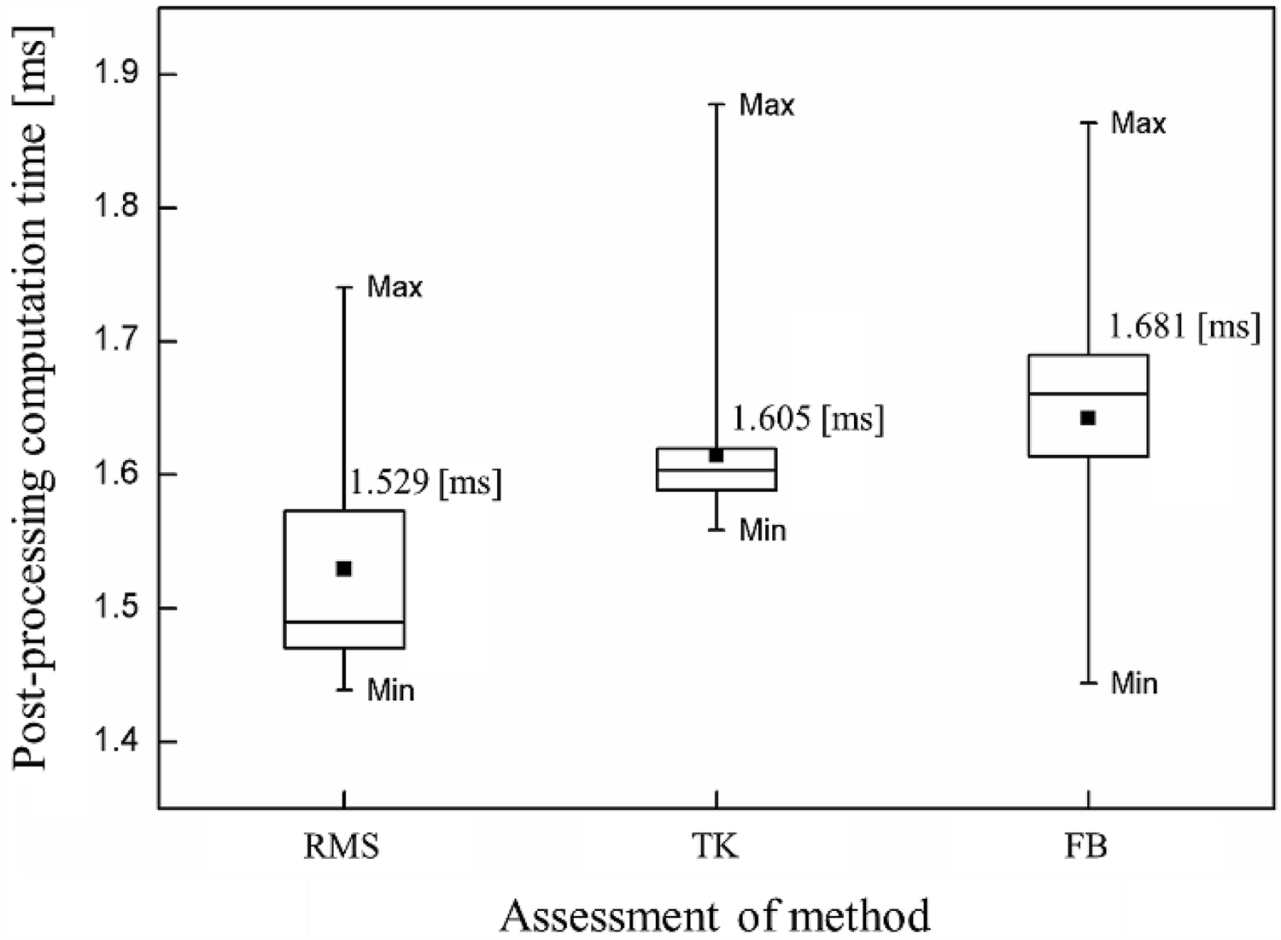
Box plot of the post-processing computation time of each CI assessment method.

Figure 12 shows the results of each t_com_ (first–third quartile with whiskers from the minimum to maximum) by performing 100 iterative calculations in consideration of the deviation of the time to load the DP signal from the library in the Python program. The t_com_ values from the RMS, TK, and FB methods are 1.529 ms, 1.605 ms, and 1.681 ms, respectively. It requires only 7.6%, 8.0%, and 8.4%, respectively, of the time to acquire the dynamic signals of one frame. Therefore, it takes much more time to acquire the DP data rather than to evaluate the combustion state or CI onset. The t_com_ value of the FB method is lower than that of the RMS and TK methods. Nevertheless, the relatively slow computation time has little effect on evaluating the combustion state and detection time of CI because it takes only 0.38% more time when compared to the data acquisition time. In fact, the detection time of CI using the FB method is up to 300 ms (15 frames) faster than the other methods. Therefore, the FB method, which is proposed in the present study, can be an effective diagnostic tool of CI for a gas turbine combustor.

## Conclusion

In this study, the FB method was introduced as a potential indicator that can detect CI in a model gas turbine combustor. Combustion experiments were carried out in a partially premixed combustor for various fuel compositions of H_2_ and CH_4_. The performance of FB together with RMS and TK were compared during the CI transition process from the perspectives of accuracy, detection time, and multi-mode detection possibility. Based on the results, we can conclude the following.

1. The CI detection time of RMS, TK, and FB are summarized in Table 7. FB and TK show the comparable performance in CI detection time with the RMS method. In terms of detection sensitivity, RMS cannot detect weak CI, but TK and FB show sensitive and accurate performance in detecting weak CI. Herein, it should be noted that the RMS method is also more sensitive if the criterion of RMS is set to be lower. Thus, it is difficult to definitively state that FB methodology is superior to the existing methodologies of RMS or TK.
2. However, a lower criterion will increase the probability of false-positive warnings of CI, so that the use of multiple precursors is recommended to enhance the robustness of prediction of onset of CI. That is, FB can complement other measures in detecting the onset of CI and, in this manner, it would allow one to avoid false-positive or even false-negative warnings.
3. TK is a signal-processing method based on a single CI frequency, but FB calculates the power spectrum in the entire frequency domain over time. Therefore, the FB method has an advantage in multi-mode CI detection as well as single-mode CI detection that usually occurs from H_2_/CH_4_ flames in a combustor.

**Table 7:** Summary of detection times using RMS, TK, and FB.

#	Case 1 → Case 2 [s]	Case 3 → Case 4	
		First peak [s]	Second peak [s]
RMS	2.33	No detection	2.63
TK	2.05	0.63	2.43
FB	2.03	0.61	2.41
